# Corruption

**DOI:** 10.1590/S1679-45082017ED4239

**Published:** 2017

**Authors:** Jacyr Pasternak

**Affiliations:** 1 Hospital Israelita Albert Einstein, São Paulo, SP, Brazil.

We all are amazed by the extent of corruption in our political system. We believe corruption is frequent or even the norm, such as to receive money from private construction companies that build for the government, exchanging favors and money for politicians. As an excuse, some entrepreneurs declared that without these favors they would never get the contracts... The corrupted politicians said that without the money given by these companies, they would be unable to pay for their election campaigns, which are increasingly more expensive. If they run to win, candidates have to pay the advertisement team and the work of these guys is not cheap at all. They will give politicians a discount if paid with slush money, or, even better, if the sum is deposited abroad.

Unfortunately, the medical practice faces similar problems. Everyone working in this field has known for many years that prostheses are chosen based on the financial support given by the manufacturers to physicians who prescribe them. Other lesser known sins, although much more frequent, are physicians paid to write fake reports for individuals not going to work, prescriptions of drugs that are not always the cheapest or best for the patient, speakers who do not disclose intimate relationships with the products they recommend during their lectures, surgeries that are not the best options for patients. This list is quite long...

The Ethical Committees at hospitals and the State Medical Boards (*Conselhos Regionais de Medicina*) will act if provoked, but essentially, physicians and other healthcare professionals are reluctant to denounce these situations. This fact is partly related to the military dictatorship we had in Brazil: we do not like whistleblowers… On the other hand, there is a false ideal of class solidarity. Furthermore, it is very difficult to deal with too much red tape – the boards are agencies of the Federal Government and work at a slow and irritating pace. This is not a Brazilian problem: many articles were published in the United States addressing issues related to whistleblowers. After denouncing, many professionals stated they would not do it if they knew the consequences, including trouble, appeals, legal matters and so on.

We understand their plight. However, if healthcare professionals do not make an effort to minimize corruption in their workplace – we say minimize, since it is impossible to put an end on corruption considering how human beings are – their reputations will be tainted likewise the Brazilian politicians. Yet, somewhere there are decent politicians who work for the common good. But try to ask any Brazilian citizen if they know such an animal, and hear what they will say… We do not want to be deemed as they are…

